# Assessment of remnant liver function and volume after selective ligation of portal vein and hepatic artery in a rat model^1^

**DOI:** 10.1590/s0102-865020190110000003

**Published:** 2020-01-10

**Authors:** Thiago Boechat de Abreu, Alexandre de Abreu Ribeiro, Lívia Paola Colchete Provenzano, Joaquim Ribeiro, Alberto Schanaider

**Affiliations:** I MSc, Department of Surgery, Faculty of Medicine, Universidade Federal do Rio de Janeiro (UFRJ), Brazil. Conception, design, intellectual and scientific content of the study; acquisition, interpretation and analysis of data; manuscript writing; critical revision; II Fellow Master degree, Department of Surgery, Faculty of Medicine, UFRJ, Rio de Janeiro-RJ, Brazil. Conception, design, intellectual and scientific content of the study; interpretation and analysis of data; critical revision; III Head and Full Professor, Department of Surgery, Faculty of Medicine, UFRJ, Rio de Janeiro-RJ, Brazil. Conception, design, intellectual and scientific content of the study; interpretation and analysis of data; critical revision; IV PhD, Assistant Professor, Department of Surgery, Faculty of Medicine, UFRJ, Rio de Janeiro-RJ, Brazil. Interpretation and analysis of data, critical revision

**Keywords:** Hepatectomy, Liver Regeneration, Liver Failure, Portal Vein, Hepatic Artery, Rats

## Abstract

**Purpose:**

To evaluate liver regeneration after selective ligation of portal vein and hepatic artery by 3D Computed Tomography in an experimental model.

**Methods:**

Sixteen Wistar rats were randomized into four equal groups: Group I- control (sham), Group II- isolated selective ligation of the hepatic artery, Group III- isolated selective ligation of the portal vein and Group IV- combined ligation of portal vein and hepatic artery. Before procedure and five days after a 3D CT Scan was performed to analyze the hypertrophy, weight and function of the remnant liver.

**Results:**

The largest regeneration rate and increase of weight in the hypertrophied lobe was detected in group IV, the first with an average of 3.99 (p=0.006) and the last varying from 6.10g to 9.64g (p=0.01). However, total liver weight and the R1 ratio (Hypertrophied Lobe Weight/Total Liver Weight) was higher in group III (P<0.001) when compared with groups I, II and IV and showed no difference between them. The immunohistochemical examination with PCNA also found higher percentages with statistical significance differences in rats of groups III and IV. It was possible to confirm a strong correlation between hypertrophied lobe weight and its imaging volumetric study. Liver function tests only showed a significant difference in serum gamma-glutamyltransferase and phosphorous.

**Conclusion:**

There is a largest liver regeneration after combined ligation of portal vein and hepatic artery and this evidence may improve the knowledge of surgical treatment of liver injuries, with a translational impact in anima nobile.

## Introduction

The first liver surgery was performed just over 100 years ago^1^. Considered a complex procedure due to the occurrence mainly of bleeding and biliary fistula, the technique underwent several modifications in this last century^1^ ^,^ ^2^. In addition to technical changes, the emergence of energy devices - as monopolar, bipolar and radiofrequency energy - and ultrasonic aspiration, allowed more extensive resections to be performed. All these advances have brought a new challenge: hepatic failure, which is the most common cause of mortality after extensive respective liver surgeries^1^ ^,^ ^4^.

Currently, the recommendation for remaining liver percentage to avoid this complication is 20% for people with normal hepatic function, 30% for patients receiving chemotherapy and 40% for patients with hepatic cirrhosis independent of the causative factor, although there is no portal hypertension^3^ ^,^ ^4^. When doing surgery implies leaving less hepatic parenchyma than recommended, it is to perform - before surgery - procedures that will promote hepatic hypertrophy. Thereby, resection is possible keeping a safe remnant.

The strategy most used today to promote hypertrophy of the remaining liver is embolization or selective ligation of the portal vein^5^ ^–^ ^11^. This procedure is usually performed one month before resective surgery and promises to increase up to 67% in non-ischemic hepatic volume^7^. Although the data are encouraging, certain patients (especially oncological patients) need faster response rates. Minding this, several studies have been developed, seeking to promote equal or superior results in the preoperative gain of liver function and volume in a shorter period of time.

One of the most prominent techniques was ALPPS (combined hepatic bipartition with portal vein ligation for two-stage hepatectomy), showing hepatic regeneration results in the order of 40-80% in 6-9 days^5^. In addition, the technique of portal embolization associated with hepatic artery ligation compromising about 70% of hepatic vascularization showed even better results of regeneration rate of 104% in 7 days^12^.

In order to better understand the mechanisms of liver regeneration in the combined portal vein and hepatic artery ligation, we reproduced the procedure in an animal model and studied the results through images obtained by 3D computed tomography with volumetric liver analysis. These images, added to hepatic function enzymes dosage, allowed to evaluate the regeneration rate of the remaining liver, to estimate its weight and functionality.

## Methods

The research was approved by the Animal Use Ethics Committee of Universidade Federal do Rio de Janeiro (UFRJ) in accordance with Brazilian legislation and international guidelines (Process number 87/09). The study was carried out at the Center of Experimental Surgery, School of Medicine – UFRJ.

Sixteen adult male Wistar rats (*Rattus norvegicus*) weighing between 220g and 290g were used. The animals were kept in individual cages, under temperature control and a 12 hour light/dark cycle at the Center of Experimental Surgery, School of Medicine – UFRJ. They received free water, standard feeding and hygiene care.

The sixteen rats were randomly distributed in four equal groups: Group I - control (sham), Group II - isolated selective ligation of the hepatic artery, Group III - isolated selective ligation of the portal vein and Group IV - combined ligation of portal vein and hepatic artery.

### Study steps and surgical procedure

All rats were undertaken to intraperitoneal anesthesia with ketamine (50mg/ml – 0.3ml) and xylazine (20mg/ml – 0.1ml) soluction injection. Under anesthesia, we performed preoperative computed tomography and then proceeded to surgery.

The procedure consisted in median laparotomy, access of the hepatic pedicle of the left medial, left lateral and right medial liver segments and the vascular ligation proposed to each group. In control group, the hepatic pedicle was just mobilized, without any ligation. The analgesia in the immediate postoperative period was performed with paracetamol drops at a concentration of 200 mg/mL diluted in 100 mL of water and offered ad libitum. These three segments correspond to some 70% of the overall liver mass.

On the fifth postoperative day, under the same anesthetic protocol, we performed a new computed tomography after catheterization by dissection of the jugular vein for contrast administration.

On the seventh day after surgery, the rats were killed, in a painless procedure, with a mixture of 2% xylazine at the dose of 40 mg/kg and 10% ketamine at the dose of 400 mg/kg intramuscularly. Death was characterized by respiratory arrest and complete absence of reflexes. Blood samples were collected from the vena cava for biochemical analysis and the liver was completely removed for examination of its segments and weighing.

### Liver regeneration evaluation

In order to evaluate hepatic regeneration, we used images obtained from Phillips ICT 256 channels scanner, tube voltage and current of 120kV and 70 mA respectively, at standard resolution (128 × 0.625mm collimation, tube rotation time of 400ms, 20 cycles, each one with gantry of 0.6s) with the following parameters: sequential acquisition of images with hepatic perfusion protocol, axial images performed with minimum time interval (0.6 seconds) and a helical extension of 7/8cm. Field of view depended on rat's size.

Reconstruction was done with standard B filter (512 × 512 image matrix with a window width of 345 to 360H, center of 80H and 0.8mm slice thickness with 0.4mm increment).

The iodinated contrast agent used was Henetix (Iobitridol, 350 mg / ml) from the Guerbet laboratory, Rio de Janeiro, Brazil. It was injected manually into previously dissected jugular access, at a dose of 0.1 ml per 100 mg of body weight. Images were obtained immediately after the end of contrast infusion.

The image analysis was done with the PACS visualization software (Carestream, v. 11.0), with a specific 3D reconstruction software package using Maximum Intensity Projection (MIP) and Volume Rendering Technique (VRT) methods, for tissue definition and characterization.

With pre and post-operative images taken the way described above and using the mentioned software, it was possible to calculate for each animal the liver regeneration rate using the following formula: Regeneration volume rate = post-surgical hepatic volume / pre-surgical hepatic volume.

Regeneration was also measured by direct weighing of the liver and its hypertrophied non-ischemic segments on a high precision scale, generating another data called R1 (ratio of hypertrophied lobe weight [HLW] and total liver weight [TLW]). It represents the percentage of hypertrophied lobes in relation to the total liver weight. So it is possible to decrease the variation attributed to natural differences between the rats and the absolute livers weight. The data obtained through the weighing were compared with those obtained in the imaging volumetric studies, with the application of statistical tests for agreement. We also used the body weighing data of all rats prior to any intervention and immediately prior to euthanasia.

### Liver function and hepatocellular lesion assessment

Prior to euthanasia, all rats had blood samples collected directly from the vena cava. They were submitted to centrifugation (10 minutes at 3000 rpm) and plasma was obtained for calculating biochemical dosages of the following elements: alanine aminotransferase (ALT), lactic dehydrogenase (LDH), direct and indirect bilirubin, phosphorus dosage, phosphatase alkaline and gamma-glutamyltransferase (gamma-GT).

### Immunohistochemistry analysis

In addition to imaging and blood studies, we performed immunohistochemical analysis of the hepatic tissue of the hypertrophied lobe. These fragments, obtained from all animals, were sliced at the thickness of 4µm and evaluated by optical microscopy at a 20-fold objective increase. Through the use of the cellular proliferation nuclear antigen (PCNA-PC10), we obtained the index of cellular proliferation expressed by the percentage of stained nuclei.

### Statistical assessment

Kruskal-Wallis non-parametric test was used to analyze the data in order to compare the numerical variables among the four groups. To calculate the “p-value” in the intergroup comparison, we used the Bonferroni correction. The simple linear regression model with intersection equal to zero, Pearson's correlation coefficient, Bland-Altman graph and t-Student test were used to evaluate the correlation between the hypertrophied lobe weight (HLW) and the post-surgical volumetric study (postVol) of this same lobe. We considered statistically significant a p-value of 0.05 (5%). The software used for statistical analysis was R 2.12.2.

## Results

There were no deaths caused by the surgical procedure, so it was possible to acquire all liver measures and laboratory parameters. Likewise, all CT tests and their reconstructions were successfully performed in all groups.

### Blood analysis

Comparative ALT, LDH and bilirrubins values in blood plasma had no statistical differences between groups. Only phosphorous and gama-GT analysis showed statistical significance differences. Phosphorous between groups I and II (p=0.03) and gama-GT between groups II and IV (P=0.05). Results are expressed in Table 1.

**Table 1:** Blood plasma biochemistry.

	PHOSPHOROUS (mg/dL)	GAMMA-GT (UI/L)
GROUP I	12..8 (4.5)	8.5 (7.0)
GROUP II	6.2 (1.6)	5 (0)
GROUP III	7.9 (1.6)	9.2 (4.8)
GROUP IV	9.2 (2.1)	19.8 (11.2)
*p-VALUE*	0.03	0.05

Results given as mean values and standard deviation (in parentheses).

### Rats weight loss assessment

**Figure 1:**
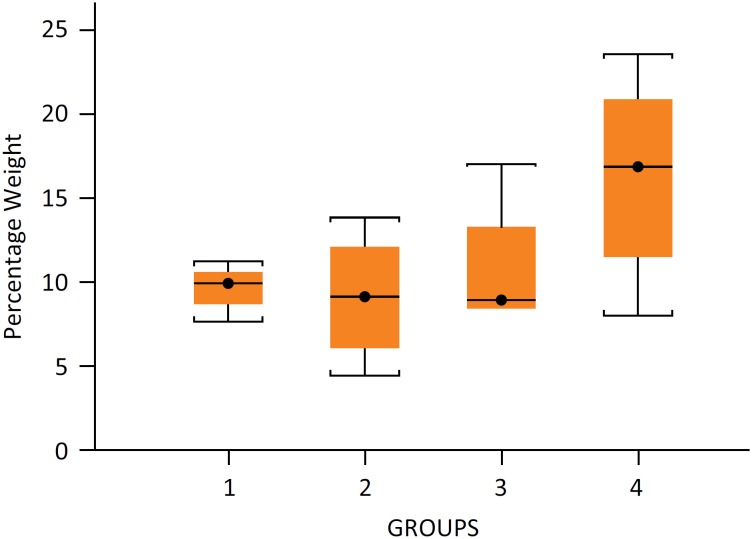
Rats weight loss percentage. Results expressed by percentual mean, minimun and maximus, in parentheses. Group I: 9.6% (7.6-11.1); Group II: 9.0% (4.4-13.7); Group III: 10.8% (8.4-17.0); Group IV: 16.2% (8.0-23.4). n=4/Group.

At the end of the study, it was possible to verify, by simple visual observation, that there was a more pronounced loss of muscular mass among rats in group IV. However, although there were differences in post-intervention weight, it was not statistically significant (p=0.3775 - group IV x group I). Data in Figure 1.

### Liver weight regeneration

**Figure 2:**
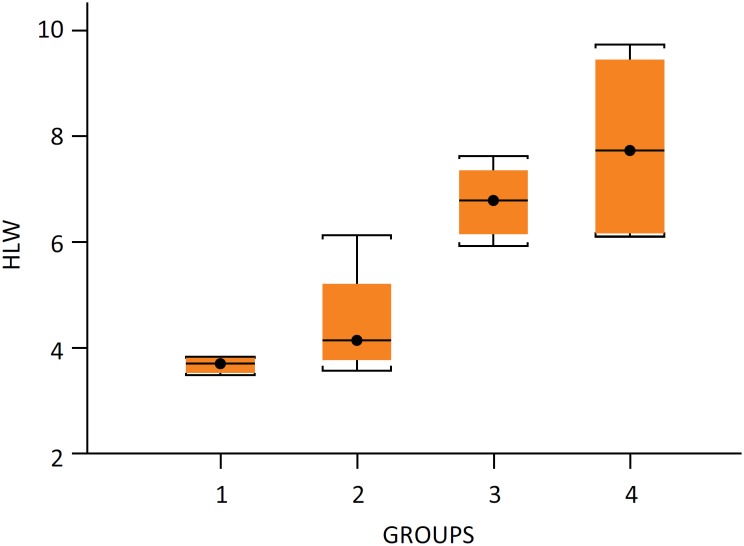
Hypertrophied lobe weight. Results given as minimum - maximum values and mean values in parentheses. Group I - 3.5g – 3.8g (3.7g), group II – 3.5g – 6.1g (4.1g), group III – 5.9g – 7.6g (6.8g) and group IV – 6.1g – 9.7g (7.6g). n=4/group.

**Figure 3:**
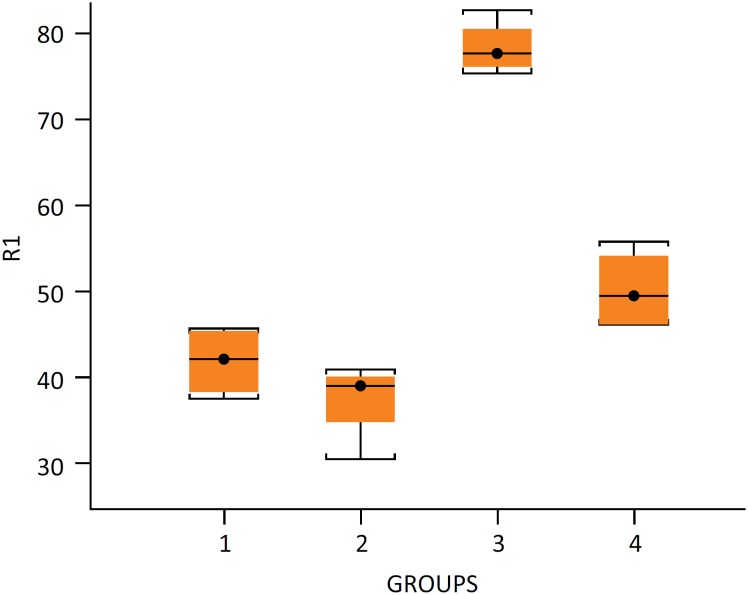
R1 ratio (Hypertrophied Lobe Weight [HLW] / Total Liver Weight [TLW]). Minimum and maximum values, mean value in parentheses: group I - 0.375 – 0.458 (0.422), group II - 0.306 – 0.409 (0.391), group III - 0.754 – 0.828 (0.776) and group IV - 0.463 – 0.558 (0.495). n=4/group

On the seventh day after surgery, the liver was completely removed for examination of its segments and weighing. There was a real hypertrophy in group III (P=0.018) and group IV (P=0.001) compared with sham group. When we compared group III with group II (P=0.041) and group IV with group II (P=0.005), there was also statistical significance. However, the groups III and IV rates showed no differences between them (P=0.68) (Fig. 2). The R1 ratio (Hypertrophied Lobe Weight/Total Liver Weight) was higher in group III (P<0.001) compared with groups I, II and IV, but showed no difference between these last three groups, as showed in Figure 3.

### Liver volumetric study regeneration

**Figure 4:**
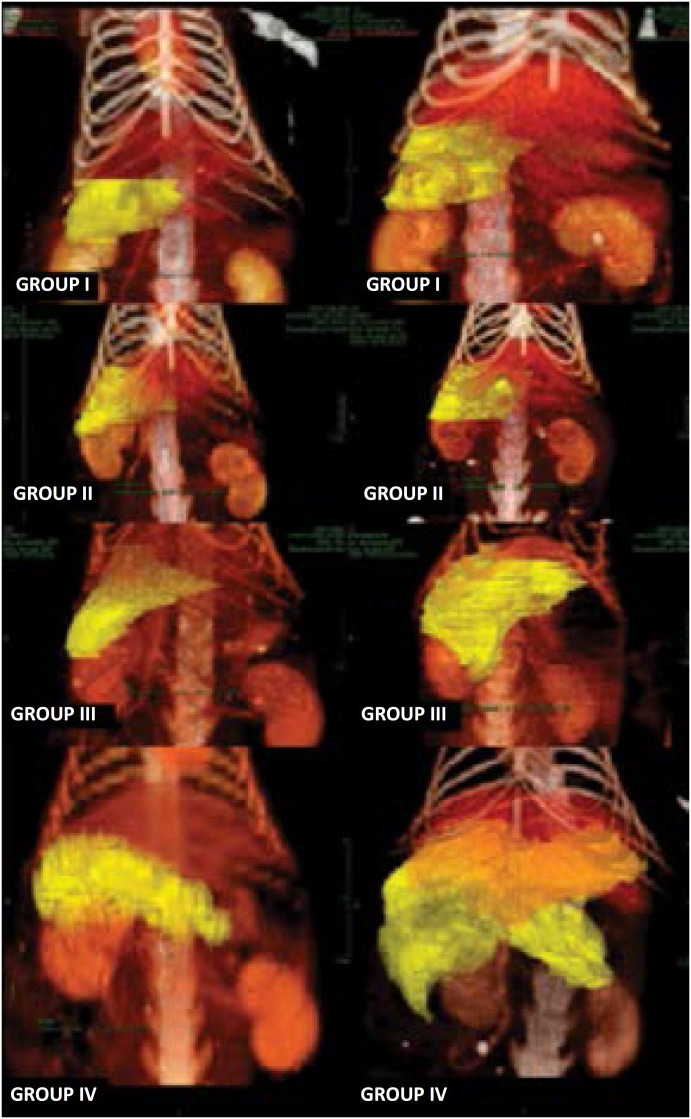
3D CT scan with volumetric reconstruction. Before surgery and 5 days after procedure. Left column: before, right column: after.

We performed 3D CT scanner images with volumetric reconstruction for all sixteen animals. Examine was performed before and after surgery (5th postoperative day). Figure 4 shows images of one rat from each group, randomly selected, comparing pre and postoperative hepatic volume. All data from post-surgical volumetric study as well as the regeneration volume ratio are showed in Table 2. In the first statistical differences were found between groups I and III (P=0.002), I and IV (P<0.001), II and III (P=0.009), II and IV (P<0.001) and also between groups III and IV (P=0.017). The second data showed difference statistically significant between groups I and III (P=0.018), I and IV (P=0.001), II and III (P=0.030), II and IV (P=0.002), but no difference was found between I and II or between III and IV. The highest regeneration volume ratio and non-ischemic lobe post-surgical volume were showed in group IV.

**Table 2:** Liver volumetric study data.

	POST-SURGICAL VOLUMETRIC STUDY (cm^3^)	REGENERATION VOLUME RATIO
GROUP I	3.21 (0.4)	1.03 (0.04)
GROUP II	3.86 (0.6)	1.18 (0.1)
GROUP III	6.33 (1.18)	3.02 (1.2)
GROUP IV	8.59 (1.07)	3.99 (1.05)
*p-VALUE*	<0,001	<0,001

Results given as volume mean values and standard deviation (in parentheses).

**Figure 5:**
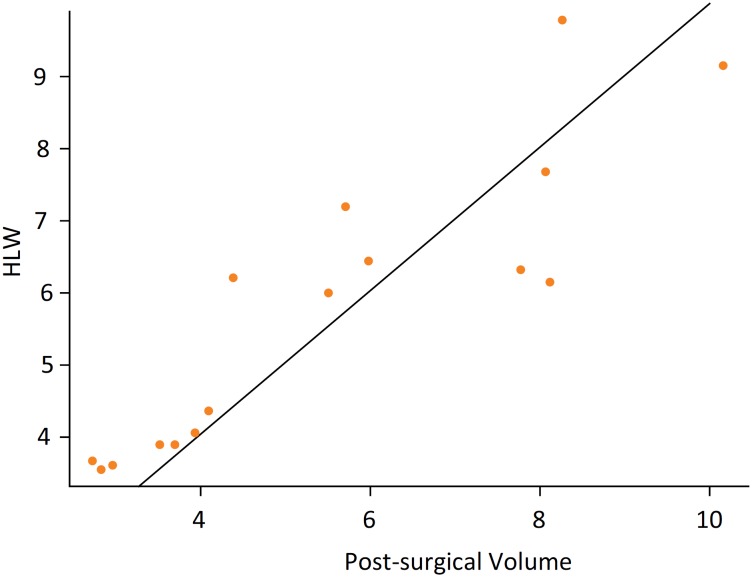
Angular coefficient. Nearly to 1, showing strong correlation between hipertrophied liver weight and post-surgical non-ischemic hepatic volume.

**Figure 6:**
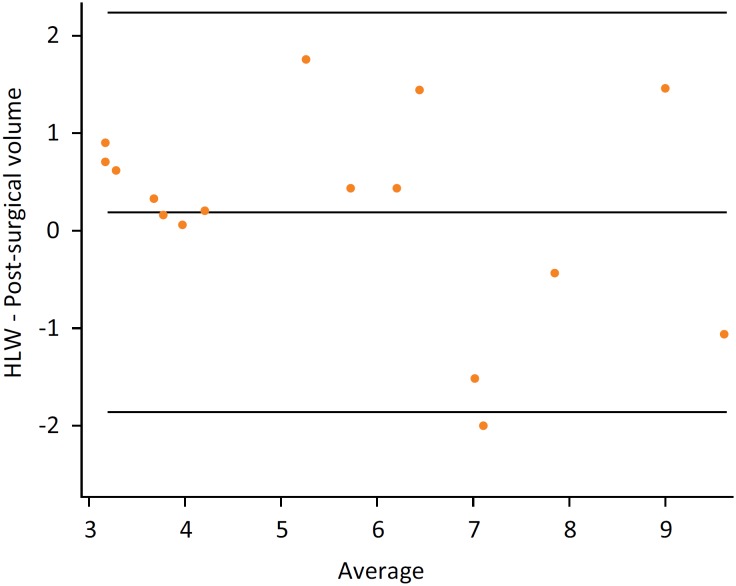
Bland-Altman. Results proves the agreement between the hypertrophied liver weight and its volume.

The volume and weight data of the hypertrophied hepatic lobe used to estimate the liver regeneration ratio were also compared to each other in order to evaluate the accuracy of imaging methods in the volumetric measurement of the liver. The results showed a strong correlation between them, shown in Figure 5, through the angular coefficient nearly to 1 (P = 0.98) and in Figure 6 where the bias was almost zero. Both analyses favor the idea of reliability of the CT Scan method in the evaluation of hepatic volumetry.

### Immunohistochemistry analysis

**Figure 7:**
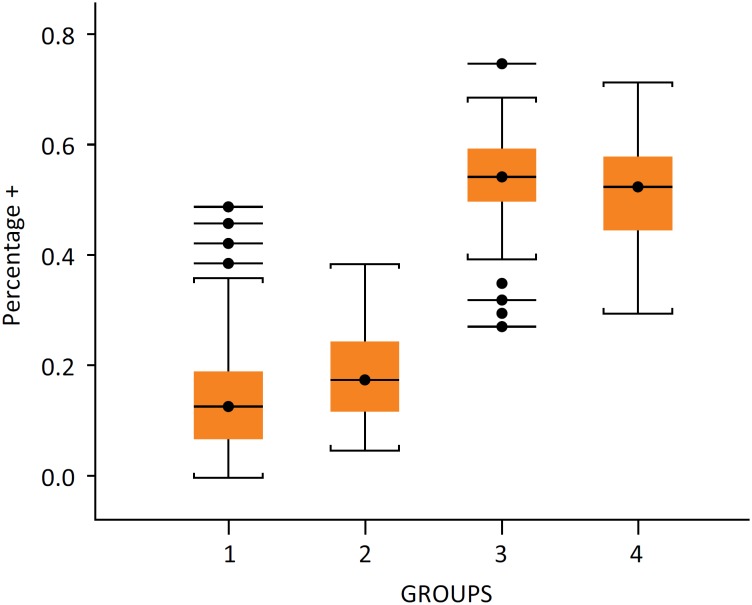
Average percentage of nuclei stained. Each group and their standard deviation in parentheses: group I – 0.149 (0.108), group II – 0.188 (0.087), group III – 0.54 (0.088) and group IV – 0.515 (0.092). n=4/group.

**Figure 8:**
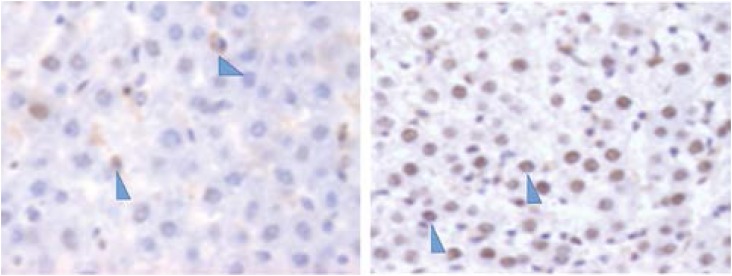
PCNA immunohistochemistry samples. Optical microscopy (x20). Left image: group I, right image: group IV. Arrows showing the brown stained nucleus cells.

Immunohistochemistry was performed in all groups to estimate cell proliferation in the hypertrophied hepatic lobe. There were statistically significant differences comparing the percentage of stained nuclei between groups I and III, I and IV, II and III, II and IV (P<0.0001). The exact positive percentual values and standard deviation are shown in Figure 7. In Figure 8, the microscopy picture is showing the differences in the amount of nuclei stained between groups.

## Discussion

### Liver surgery, past and evidences

Hepatectomy continues to be the most effective treatment in malignant liver tumors such as hepatocarcinoma, intra-hepatic cholangiocarcinoma and metastatic liver lesions^13^. Recent advances in surgical techniques and procedures, as well as in pre-surgical, surgical and post-surgical care, have made liver resections safer. However, lethal complications, especially due to liver failure after surgery are still a challenge on wide resection. Therefore, it is imperative to improve techniques in order to increase the remnant liver volume. Currently, remnant liver volume assessment is a fundamental part of surgical planning for patients who are good candidates for healing resection^14^ ^,^ ^15^.

### Liver regeneration due to vascular procedures

In this study, undertaken with vascular ligation, average rates of regeneration of 302% and of 399% were obtained in groups III and IV, respectivelly (Table 2), with hypertrophied liver increasing in group III and group IV, reaching 78% and 50% of the overall liver weight, respectively (R1 rate in Figure 3), in seven days. Literature data confirm such findings, as presented by Sugimoto *et al*.^16^. Liu and Zhu^9^ performed portal vein embolization and have shown regeneration rates of 20% and 46%, within 2 and 8 weeks respectively, in remnant liver volume. Abdalla and Vauthey^7^ using the same technique have found a parenchymal gain ranging from 31 to 59% of the overall liver volume, after embolization.

Our study showed real gain in hypertrophy (absolute weight in grams) in isolated portal vein ligation group and when an arterial ligation was associated, with a non-ischemic liver segments weight in groups III, ranging from 5.96g to 7.64g, and group IV, ranging from 6.11g to 9.74g (Fig. 2). The comparison between the percentage of the hypertrophied segment with the overall liver weight (R1 ratio rate), was significant only in group III, ranging from 75.4% to 82.5% (Fig. 3, P=0.0045). In group IV, there was no significant difference in R1 when compared to the Sham group. This finding indicates that, in group IV, the ischemic liver parenchyma did not reduce as much as in group III, influencing the percentage that the weight of the hypertrophied lobe represents in the total liver. Thus, although the selective portal vein associated with hepatic artery ligation shows a higher liver hypertrophy in group IV, it suggests an impair of the animal overall status, demanding further studies to assess the procedure safety level. These results allow to conclude that if in addition of portal vein ligation a hepatic artery ligation is performed, the largest absolute gain in liver mass can be achieved. Moreover, if it is transposed to a clinical practice, may transform a patient who is not a candidate to wide liver resection into one who is a candidate to that high-healing-probability procedure.

### Laboratorial findings and immunohistochemistry analysis

As blood plasma markers were tested only at the end of the study, i.e., on the seventh day of ischemia, if there was any sign of transient hepatocellular damage, we did not evaluate it. It means that in neither group the procedure led to severe or permanent metabolic insufficiency, as seen in biochemical tests. Hypophosphatemia presented in groups II, III and IV suggests adequate hepatic regeneration. Even in group IV, with significant parenchymal ischemia, the remaining liver was able to fulfill its metabolic functions, but the increase of serum gama-GT in this group, as a marker of oxidative stress, may indicate a greater process of inflammation in the extracellular liver microenvironment^17^ ^–^ ^21^. Comparing our results on the seventh day of the experiment with those obtained in several other studies, similar facts can be found with ALT and LDH, even in different animal species (rats, rabbits and pigs)^22^ ^–^ ^25^. However, they showed an increase of such parameters in the first two days with a peak at 72 hours, descending and then normalizing on the seventh day. Likewise, we did not find any significant repercussions in relation to the percentage of weight loss, although macroscopically the animals in group IV presented higher muscle mass consumption.

PCNA plays a crucial role in DNA replication and its expression is related to cell proliferation^26^. The high percentage of nuclei stained with PCNA in groups III and IV showed intense cellular proliferation after the intervention when compared to the sham group. This finding reinforces the hypothesis that there was a higher rate of hypertrophy in the animals submitted to ligation of the portal vein and portal vein associated with arterial ligation, when compared with groups I and II.

### Image assessment

As seen, CT with 3D reconstruction and volumetric study of remnant liver is an important test for a successful two-stage surgical strategy or any broad resection of the liver^27^ ^,^ ^28^. The only experimental study that performed such procedure was recently published by Van den Esschert *et al*.^29^, using rabbits, demonstrated a mean gain in the caudate lobe of 15% ±4% for PVL technique and 22% ±2% for PVE technique, after 14 days. We did not find any other study in rats using a high-resolution CT scanner, 256-channel, with high-speed image acquisition and processing capability, performing reconstruction. This allowed us to apply 3D liver reconstruction techniques to obtain the volume of the desired segments and to evaluate the regeneration rate for each animal studied. Statistical tests indicated that the volumetric study was able to measure the mass of the hypertrophied segments with complete confidence. The model confirms the correlation between weight and 3D CT with volumetric reconstruction and its validation found in this paper indicates new paths in other studies of liver size measurement. We can affirm that animals in groups III and IV were the ones that reached the highest regenerate volume ratio, and that the absolute post-surgical liver remnant volume showed a greater hypertrophy in group IV, when compared to any other group.

### Final considerations

The results of this study had some limitations because of the number of animals in each group due to temporary restrictions on the use of animals in surveys by inspection agencies. A new study, with larger groups, would be necessary, further to subgroups with different euthanasia times to assess the evolution of each variable, over time. In this way, information produced can improve knowledge in the field of liver surgery.

## Conclusions

The acquisition of liver images with 3D CT reconstruction proved to be a reliable method for measuring the volume of hypertrophied hepatic segments after surgery. Evidence of rapid and major hepatic regeneration without functional impairment after a 70% restriction of liver blood flow due to combined ligation of portal vein and hepatic artery may improve surgical treatment of liver lesions.
